# Trajectories and risk factors for anxiety and depression in children and adolescents with cancer: A 1‐year follow‐up

**DOI:** 10.1002/cam4.4100

**Published:** 2021-07-26

**Authors:** Maya Yardeni, Gadi Abebe Campino, Ilanit Hasson‐Ohayon, Dana Basel, Nimrod Hertz‐Palmor, Shira Bursztyn, Hana Weisman, Itai M. Pessach, Amos Toren, Doron Gothelf

**Affiliations:** ^1^ The Chaim Sheba Medical Center Ramat Gan Israel; ^2^ Sackler Faculty of Medicine Tel Aviv University Tel Aviv Israel; ^3^ Sagol School of Neuroscience Tel Aviv University Tel Aviv Israel; ^4^ Department of Psychology Bar‐Ilan University Ramat Gan Israel

**Keywords:** anxiety, cancer, depression, longitudinal, PROMIS, risk‐factors

## Abstract

**BACKGROUND:**

There is limited data on the longitudinal trajectories of psychiatric disorders in children with cancer and risk factors for their persistence. The current study aimed to longitudinally assess the trajectories and risk factors for anxiety and depressive symptoms and disorders in children and adolescents with cancer.

**METHODS:**

Children and adolescents with cancer and their parents completed the Patient‐Reported Outcomes Measurement Information System (PROMIS) Depression and Anxiety Module and were interviewed by the semi‐structured Affective and Anxiety Modules of the Kiddie Schedule for Affective Disorders and Schizophrenia for School‐Age Children (K‐SADS), at 4 time points, 1, 4, 7, and 12 months following the diagnosis of cancer.

**RESULTS:**

Of the 99 patients enrolled, 48% met criteria for anxiety and/or depressive disorders at least once during the follow‐up period. There was a significant decrease in PROMIS pediatric and parent anxiety and depression scores (all *p*'s < 0.01) and in the rate of depressive disorders over time (*p *= 0.02), while rates of anxiety disorders remained stable. Anxiety PROMIS pediatric and parent scores at baseline, having brain tumors and being in the acute treatment phase significantly predicted the presences of anxiety disorders at endpoint.

**CONCLUSIONS:**

Our results highlight the importance of screening for anxiety and disorders in children with cancer, especially among those with brain tumors and at the acute phase of treatment.

## Graphical abstract

This study aimed to longitudinally assess the trajectories and risk factors for anxiety and depressive symptoms in children and adolescents with cancer. We used a unique model of assessment combining the K‐SADS structured psychiatric interview along with the short PROMIS self and parent report at four time points during one year. Our results highlight the importance of screening for anxiety and disorders in children with cancer, especially among those with brain tumors and at the acute phase of treatment.

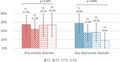

## 1 INTRODUCTION

Emotional distress is an indicator of suffering as well as a predictor of poor health and negative quality of life among children and adolescents with cancer.^1^, ^2^, ^3^ The most common forms of emotional distress are depressive and anxiety disorders that affect about 25% to 35% of this population.^4^, ^5^, ^6^, ^7^ Similar rates were found in a previous study demonstrating that in cancer patients aged 7–21 years 37.4% met the DSM‐5 criteria for depressive and/or anxiety disorders.^4^ In light of the high rates of anxiety and depression among children with cancer, the American College of Surgeons Commission on Cancer, the Institute of Medicine, the American Cancer Society, and the National Comprehensive Cancer Network require that cancer treatment centers implement screening programs for psychosocial distress of patients as a new criterion for their clinical accreditation.^8^, ^9^ Moreover, the Canadian Strategy for Cancer Control supported the proposition that emotional distress be considered the sixth vital sign that should be routinely measured in all cancer patients.^10^

Despite the wealth of data showing that depressive and anxiety disorders are common among pediatric cancer patients,^1^, ^5^, ^6^, ^7^ detailed data as to the course and risk factors of these disorders are limited. Detecting those children with cancer that are at a higher risk for prolonged distress by studying the development and risk factors of the associated disorders is crucial and will allow appropriate and timely initiation of psychiatric and psychological interventions.

The few studies that longitudinally examined the trajectories of depression and anxiety in children with cancer show inconclusive results regarding changes in symptom severity. Some of these studies used depression and anxiety questionnaires,^2^, ^3^, ^8^, ^9^, ^10^, ^11^ while others used health‐related quality of life measurements.^11^, ^12^, ^13^, ^14^ However, all longitudinal studies reported to date were limited since they relied on self‐reported or parents‐reported questionnaires for the evaluation of symptoms and did not use structured psychiatric interviews to diagnose anxiety and depressive disorders.

In most studies, the length of follow‐up was one month following diagnosis or less.^4^, ^5^, ^6^, ^7^ We found few previous studies with longer follow‐ups. One of them, with a 6‐month follow‐up from diagnosis, did not demonstrate changes in the levels of depression and anxiety symptoms over time, while another study with a follow‐up period of 1 year showed high anxiety and depressive scores 1 month after cancer diagnosis, which declined significantly at 1 year following diagnosis.^8^, ^13^

Another limitation of existing literature is that only a small number of studies examined the risk factors associated with depression and anxiety symptoms in children and adolescents with cancer. Therefore, the current knowledge about risk factors is mostly based on cross‐sectional studies. One study has shown that the levels of anxiety and depressive symptoms at 1 month after diagnosis predicted levels of anxiety and depressive symptoms at 1 year after diagnosis.^8^ Furthermore, depression and anxiety scores were found to be significantly higher during the active medical treatment phase as compared to the period post treatment.^11^

The current study aimed to longitudinally assess the trajectories of depression and anxiety symptoms and disorders as well as to assess their risk factors in children and adolescents with different types of cancer, at four time points up to 12 months following diagnosis, by using both self‐report questionnaires and semi‐structured clinical interviews. Socio‐demographic, diagnosis, and treatment‐related variables, as well as baseline anxiety and depression were examined as potential risk factors. Based on previous studies, we hypothesized that having higher baseline levels of depression and anxiety, female gender,^10^, ^12^, ^15^ older age,^10^, ^12^, ^15^, ^16^, ^17^ and having brain tumors^18^, ^19^ would predict higher levels of anxiety and depression at endpoint evaluation.

## 2 MATERIALS AND METHODS

### 2.1 Participants

The study included consecutive newly diagnosed or relapsed cancer patients aged 7–21 years treated at the Pediatric Hemato‐oncology Department, Safra Children's Hospital, Sheba Medical Center, a tertiary hospital at the center of Israel between January 2017 and June 2019. Patients without a good level of Hebrew language comprehension and /or were not capable of completing self‐assessment scales and respond to a semi‐structured interview were excluded.

### 2.2 Assessment

#### 2.2.1 Patient‐Reported Outcomes Measurement Information System (PROMIS*)*

We administered the eight‐item version of the depression and anxiety domains of the PROMIS Pediatric and Parent proxy1.1 Short Forms.^1^ The questions pertained to the previous two weeks in order to comply with the time frame of the affective and anxiety modules of the Kiddie Affective Disorders and Schizophrenia for School‐Age Children (K‐SADS) semi‐structured interview.^18^ Self‐ and parents‐reported versions of the PROMIS were used. PROMIS domains were translated into Hebrew by the standard procedure of forward‐translation and back‐translation by bilingual mental health professionals.^4^

### 2.3 The psychiatric interview

Structured psychiatric assessments were conducted through semi‐structured interviews with the participants and their parents by using the DSM‐5 Hebrew version of the affective and anxiety modules of the K‐SADS as previously described.^4^ The first part of the interview consisted of questions designed to screen for the presence of symptoms. If those questions scored above threshold for any depressive and/or anxiety symptoms, the interviewer completed the diagnostic supplements that covered the complete K‐SADS list of symptoms for depressive and anxiety disorders. These diagnoses included major depressive episode, dysthymia, generalized anxiety disorder (GAD), separation anxiety disorder, panic disorder, agoraphobia, social anxiety disorder, and specific phobias. We also included the diagnosis of a minor depressive episode when the participant had only two to four symptoms of depression for at least 2 weeks, with “mood” always being one of them, and causing significant distress or impairment in important areas of functioning.^20^

### 2.4 Socioeconomic status

The family's socioeconomic status was classified by the Israel Index of Deprivation, as published by the Central Bureau of Statistics in Israel. This is based on household census data reflecting eight aspects of material and social deprivation, dividing Israel into 20 clusters (scored 1–20, with 1 being the lowest) by residential address.^21^, ^22^

### 2.5 Procedure

The Institutional Review Board of Sheba Medical Center approved the research protocol (IRS#SMC‐3420–16). Informed consent was obtained from adult participants and from all parents. For participants under 18 years of age, informed consent was obtained from parents and assent from the participants. After obtaining consent from the participants and their parents, the child and one of his/her parents completed the PROMIS Depression and Anxiety Screening Modules at four time points, on 1 (T1), 4 (T2), 7 (T3), and 12 (T4) months following the diagnosis of cancer. After completing the questionnaires, participants and their parents were independently inteviewed using the Affective and Anxiety Modules of the K‐SADS. The assessments were conducted by trained child psychologists and social workers, who were blinded to the response of the children and parents on the PROMIS questionnaire while administering the K‐SADS. As instructed by the K‐SADS administration guidelines. In line with the K‐SADS administration guidelines, children under 14 years old were interviewed after their parent.^23^ The DSM‐5 diagnoses of depressive and anxiety disorders were determined after reaching consensus by two senior clinicians with expertise in youngsters with medical conditions (authors MY and DG).

Children identified in the interview as fullfiling DSM‐5 diagnostic criteria for a moderate to severe depressive or anxiety disorders were referred as part of the standard of care for a psychiatric evaluation and recommendations for treatment. Pertinent demographic, clinical and medical data, including age, religious affiliation, place of residence, cancer diagnoses, and history of treatment and medications were obtained from the medical record.

### 2.6 Data analysis

Analyses were performed using IBM SPSS (version 25). Levels of depression and anxiety symptoms along the 4 time‐points assessments were compared using repeated measures ANOVAs, with each of the following measures: (a) Depression PROMIS child report; (b) Depression PROMIS parent proxy report; (c) Anxiety PROMIS child report; (d) Anxiety PROMIS proxy report, with time being the within‐subject independent factor. Planned contrasts were performed to assess differences in outcomes between different time‐points (i.e., T1 vs. T2, T2 vs. T3, etc.). Non‐parametric Friedman's test was used to calculate differences in the presence of any depression and anxiety diagnoses among the time points.

For the longitudinal prediction of the presence of anxiety and depressive disorders at last participation point (T3 or T4), we conducted two hierarchical logistic regressions. The dependent variables in each of the two logistic regression were any diagnosis of anxiety or depression at last assessment point. At step one, the predictors entered were PROMIS child and parent proxy report T scores at T1. At step two, we entered all other potential predictors, including age, sex, socio‐economic status (SES), religiosity (orthodox/non‐orthodox), first cancer diagnosis vs. relapse, treatment stage at last participation point (acute/post‐treatment) and cancer diagnosis)brain vs. other tumors). Only variables with a P value of ≤0.2 were included in the final models. Participants who were not evaluated with the structured psychiatric assessment or who dropped out of the study before T3 were not included in the analysis.

## 3 RESULTS

**FIGURE 1:**
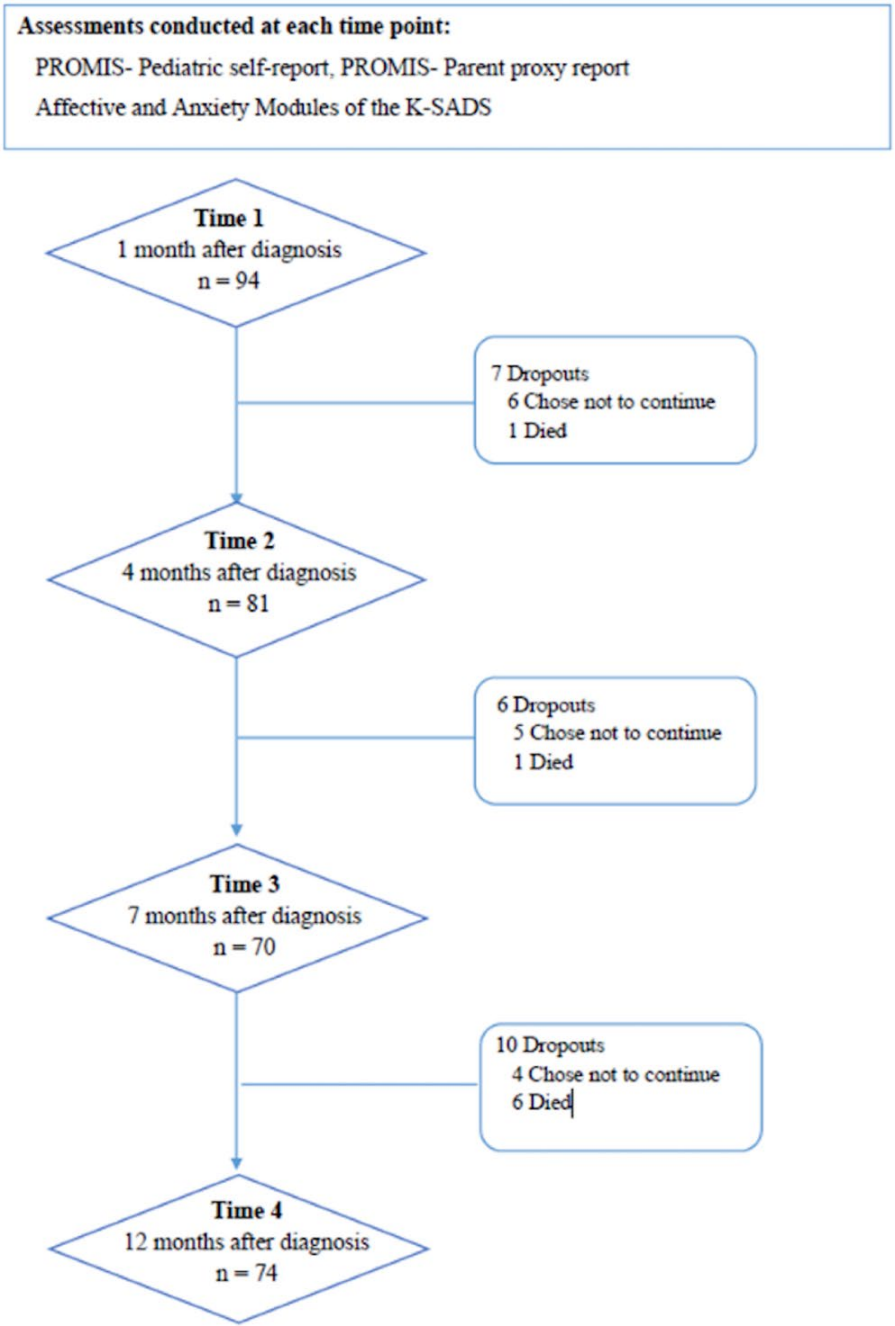
Flowchart of patients recruitment and assessment presenting the number of patients assessed at each time point and the number of dropouts. Of note, 5 patients enrolled to the study at Time 2. Due to medical travel or no‐shows some patients were not available at certain time‐points: at Time 2 (*n *= 11), at Time 3 (*n *= 15) and at Time 4 (*n *= 4)

The study included 99 participants. The flowchart depicting the study recruitment and assessment is shown in Figure 1. The demographic and clinical characteristics of the study sample are presented in Table 1 and rates of depressive and anxiety disorders at each time point are presented in Table 2.

**TABLE 1:** Demographic and clinical characteristics of the study sample (*n *= 99)

Age, mean±SD, (range)	13.56±3.63, (7–21)
Males/females, n (%)	63 (63.6)/36 (36.4)
Religious status, n (%)	
Not religious	63 (63.6)
Ultra‐Orthodox	36 (36.4)
Socioeconomic status, n (%)	
1–5	41 (41.4)
6–9	58 (58.6)
First diagnosis/relapse, n (%)	87 (87.9)/12 (12.1)
Diagnosis Group, n (%)	
Lymphoma	33 (33.3)
Sarcoma	21 (21.2)
Brain tumor	18 (18.2)	
Leukemia	16 (16.2)	
Rare tumors	11 (11.1)	
Reporting parent, n (%)		
Mother	62 (62.6)	
Father	26 (26.3)	
Other	6 (6.1)	
No parent	5 (5.1)	
Treatment stage at last participation point (T3/T4)	26 (35.1)/48 (64.9)	

**TABLE 2:** Rates of depressive and anxiety disorders at each time‐point

	T1	T2	T3	T4
	(*n* = 91)	(*n *= 76)	(*n * **= **61**)**	(*n *= 67)
Psychiatric diagnosis,				
n (%)				
Any diagnosis (depression or anxiety)	29 (39.7)	22 (36.7)	23 (31.5)	19 (28.4)
Depression and anxiety	12 (16.4)	6 (8.2)	5 (8.3)	5 (7.5)
Any depression	21 (28.8)	13 (17.8)	11 (18.3)	6 (9.0)
Major depressive disorder	11 (15.1)	7 (9.6)	6 (10.0)	3 (4.5)
Dysthymia	10 (13.7)	6 (8.2)	5 (8.3)	3 (4.5)
Any anxiety	20 (27.4)	16 (21.9)	16 (26.7)	18 (26.9)
GAD	10 (13.7)	8 (11.0)	10 (16.7)	13 (19.4)
Agoraphobia	0 (0)	0 (0)	1 (1.7)	1 (1.5)
Separation anxiety	2 (2.7)	3 (4.1)	1 (1.7)	0 (0)
Social anxiety disorder	7 (9.6)	6 (8.2)	5 (8.3)	6 (9.0)
Panic disorder	2 (2.7)	2 (2.7)	4 (6.7)	2 (3.0)
Specific phobia	4 (5.5)	1 (1.4)	1 (1.7)	0 (0)

**FIGURE 2:**
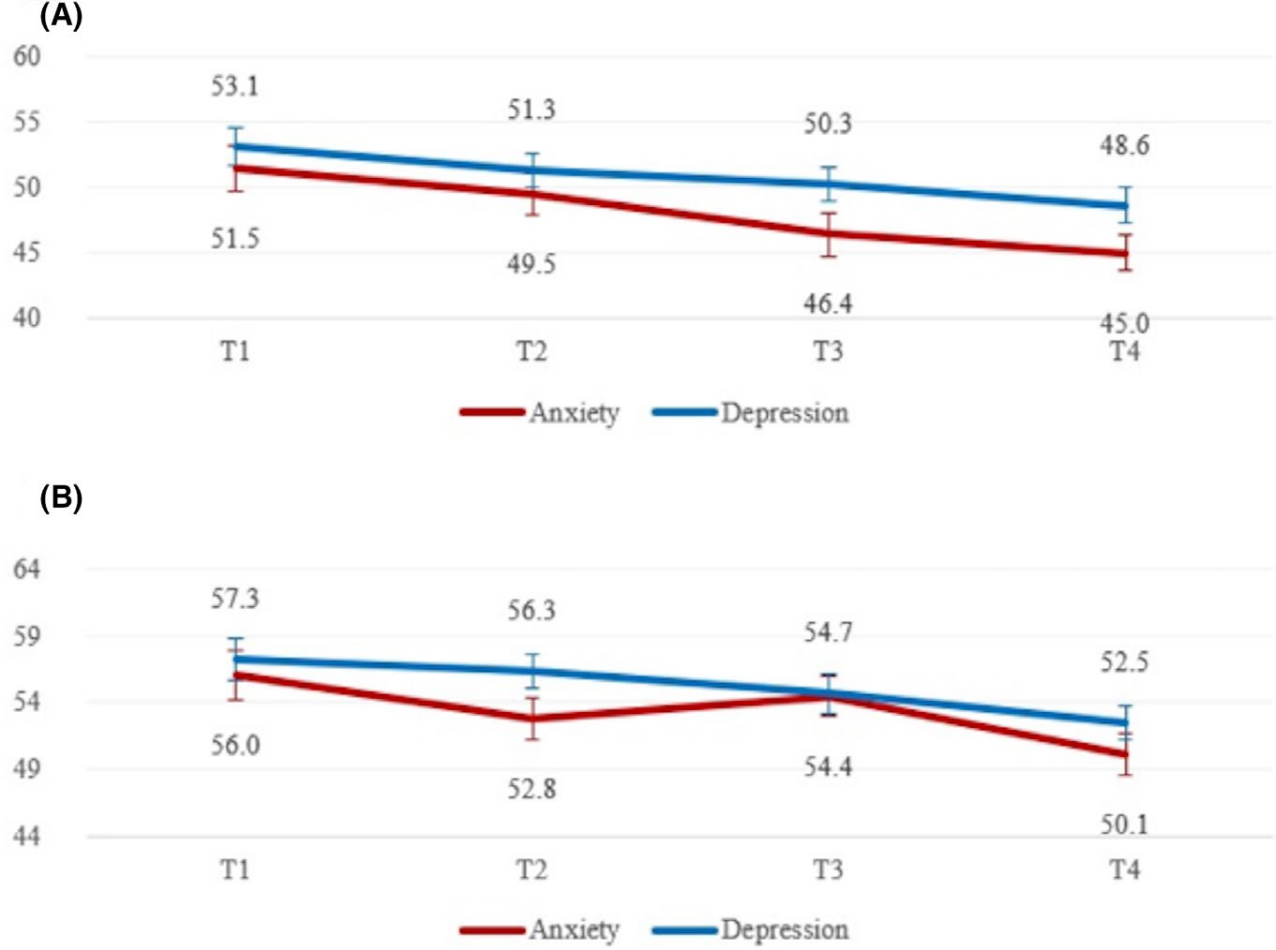
Changes in PROMIS anxiety and depression scores overtime based on (A) child report (B) parent report. Error bars represent standard errors. Planned contrasts indicated that on anxiety child report T1>T3 (*p *< 0.006); on anxiety parent report T1 > T2 (*p *< 0.039) and T3 > T4 (*p *< 0.0001) and on depression child report T1 > T3 (*p *< 0.002) and on depression parent report T1 > T4 (*p *< 0.002)

A one‐way repeated‐measures ANOVA indicated significant gradual decrease in symptoms overtime, with a linear trend in mean T scores of all PROMIS measures along the four time points (Figure 2): anxiety pediatric [*F* (2, 105) =8.32, *p*< 0.001], anxiety parent report [*F* (2, 97) =6.89, *p*< 0.001], depression pediatric [*F* (3, 132) =3.95, *p*= 0.010], depression parent report [*F* (3, 123) =4.70, *p* = 0.004].

**FIGURE 3:**
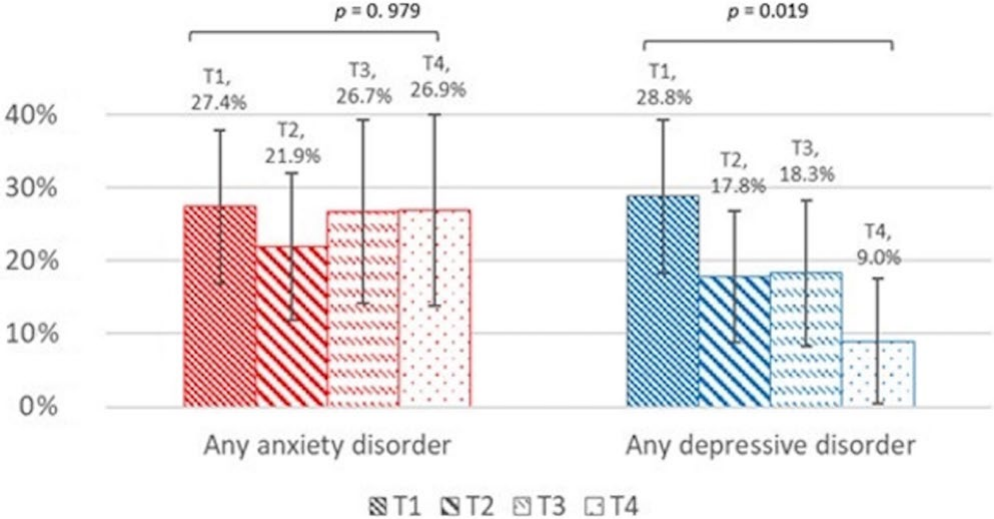
Change in rates of any anxiety and depressive disorders along time. Error bars represent 95% confidence interval. P‐values are derived from Friedman's *χ* ^2^ test

Significant differences in the rates of depressive disorders were found between time points *(Friedman's* χ^2^ (3) = 9.93, *p* = 0.019), indicating a decrease in the proportion of any diagnosis of depressive disorders over time. No significant differences between time‐points in diagnosis of anxiety disorders were found (see Figure 3).

Logistic regression analysis indicated that Anxiety PROMIS child and parent report scores at T1 were significantly associated with the presence of any diagnosis of anxiety at last assessment point (*β* = 2.04, *p* = 0.014 and *β* = 2.11, *p* = 0.019, respectively). At the second step, diagnosis of brain tumor and being in the acute phase of treatment at last participation point, were significantly associated with the presence of any diagnosis of anxiety disorder (*β* = 7.35, *p* = 0.027; *β* = 2.98, *p* = 0.014, respectively). None of the variables predicted the presence of any diagnosis of depression at last assessment point (Table 3).

**TABLE 3:** Hierarchical logistic regression with any diagnosis of anxiety at last visit as dependent variable (*n* = 74)

	DEPENDENT VARIABLE: Any diagnosis of anxiety at last participation point					
	STEP I			STEP II		
	OR	95% CI	*p*	OR	95% CI	*p*
STEP I						
Anxiety PROMIS parent at T1	2.11	1.13, 3.96	0.019*	2.22	1.14, 4.34	0.019
Anxiety PROMIS pediatric at T1	2.04	1.09, 3.80	0.025*	2.32	1.19, 44.53	0.014
STEP II						
Brain tumor				7.35	1.24, 43.52	0.027
Treatment phase at last assessment (acute/post‐treatment)				2.97	1.24, 7.12	0.014
Socio‐Economic Status				1.93	0.47, 7.98	0.362

During the longitudinal evaluation, 48 participants (48%.4) met the DSM‐5 criteria for depressive or anxiety disorders, on at least one time point assessment (Table 2). Overall, 31 patients were further referred to psychiatric evaluation, 19 underwent psychiatric evaluation, pharmacologic treatment was recommended to 17 patients out of which 11 eventually started such treatment. Five subjects were treated with serotonin specific reuptake inhibitors [fluvoxamine (*n* = 2), escitalopram (*n* = 2) and sertraline (*n* = 1)]; 5 subjects with an atypical antipsychotic medication [risperidone (*n* = 3), olanzapine (*n* = 1), and aripiprazole (*n* = 1)]; 3 with a benzodiazepine [clonazepam (*n* = 2) and lorazepam (*n* = 1)] and 1 subject received venlafaxine.

## 4 DISCUSSION

This is among the few long‐term longitudinal studies evaluating anxiety and depression in children and adolescents with cancer and the only one that applied structured psychiatric interviews, in addition to parent and pediatric PROMIS questionnaires. We found high rates of both anxiety and depressive disorders as early as one‐month following diagnosis of cancer, and a decrease in the rates of depressive, but not of anxiety disorders during the 1‐year follow‐up. Of note, a significant decrease was observed in both anxiety and depressive symptoms over time when assessed by self‐report measures, but according to the K‐SADS clinician's assessment, rates of anxiety disorders remained high. We found that baseline levels of anxiety (reported by both the children and their parents), being in an acute stage of medical treatment and having a brain tumor predicted the presence of anxiety disorders at the end of follow‐up.

Our findings of high rates (48%) of depression and anxiety disorders among children and adolescences diagnosed with cancer, at least one time point during the first year after diagnosis, are similar to those reported by previous studies.^4^, ^5^, ^6^, ^7^ In addition, decrease over time in both depression and anxiety were also shown in two studies that used self‐report questionnaires. Taken together, it seems that children and adolescents with cancer experience subjective improvement in these symptoms, possibly due to an adaptation process that takes place in which the children and their families learn to cope with the illness and its implications on their life.^9^, ^24^ However, based on our clinical assessment using the K‐SADS, we found that rates of anxiety disorders remain high, with 26.9% of our sample meeting the diagnostic criteria for anxiety disorders, 1 year following cancer diagnosis. The persistence of high levels of anxiety disorders, may be due to the fact that while approaching medical treatment completion, patients experience fear of cancer recurrence.^25^ Another possible explanation may be the complexity that accompanies the adaptation process, of returning to daily activity routine, such as returning to school and resuming social relations with peers,^15^ which may provoke anxiety.

We identified several risk factors for anxiety in children with cancer. In line with a previous longitudinal study in children with acute lymphoblastic leukemia,^8^ we found that baseline child and parent‐proxy reports of anxiety, predicted the presence of anxiety diagnosis at last follow‐up point. This finding suggests that special attention should be given to those children who experience high anxiety at baseline, as they are more likely to persist with high levels of anxiety. Additional risk factors for anxiety that were identified were having a brain tumor and being in an acute stage of treatment at the last time point of assessment. Similar to our finding, having a brain tumor was a consistent variable related with poor quality of life in children with cancer in a previously published systemic review.^26^ It is our clinical experience that children and adolescents with brain tumors often feel anxious when confronted with the cognitive and academic challenges and that this anxiety is usually more severe in children with brain tumors compared to other tumors.^18^, ^19^ Our findings, regarding the association between the acute phase of treatment and the presence of anxiety disorders, are also in line with previous studies reporting that the acute phase of treatment, consisting of aversive side effects and uncertainty regarding the success of treatment, is anxiety‐provoking.^11^, ^14^

It is important to note that we found relatively low levels of depressive disorders (9%) a year following diagnosis and therefore also failed to identify risk factors for depressive disorders. Contrary to previous studies, our study did not find age,^10^, ^16^, ^17^ sex,^10^ and sociodemographic factors^26^, ^27^ as significant risk factors for anxiety nor for depression at one year following cancer diagnosis. However, previous studies looking at risk factors for depression and anxiety in children with cancer were mostly cross‐sectional^6^, ^16^, ^17^, ^27^ and the few longitudinal studies consisted of smaller samples then our study.^3^, ^10^, ^12^

While considering the current study findings, few limitations should be considered. First, our study sample included a heterogeneous group of patients in terms of type of cancer and accompanied treatment protocols. In addition, our study is from a single institution, and included only Hebrew speakers that were able to comply with the study design, limiting generalizability of findings. Personality‐related risk factors such as coping strategies and emotional regulation abilities were not examined, limiting the studied risk factors to psychiatric, sociodemographic, and cancer diagnosis‐related variables. Lastly, time of follow‐up was limited to one year.

With these limitations in mind, the current study suggests that clinicians should pay special attention to children who express high level of anxiety at diagnosis, who cope with the diagnosis of brain tumor and whose treatment is prolonged. Our current and previous studies highlight the need to use standardized assessment of depression and anxiety, in addition to the use of short and applicable self‐reporting tools, along the disease course to facilitate early diagnosis and treatment. Interventions to increase sense of control and regulation of feelings of anxiety, such as mindfulness‐based stress reduction and cognitive behavioral therapeutic approaches early in the course of treatment may help to mitigate long‐term emotional distress.^7^ Future studies covering a larger sample size will allow further comparison of groups of children with different diagnoses and treatments as well as trace possible unique trajectories and risk factors.

## CONFLICTS OF INTEREST

The authors have no conflict of interest to declare.

## AUTHOR CONTRIBUTIONS

All authors made substantial contributions to conception and design, acquisition of data, or analysis and interpretation of data. All were involved in drafting the article or revising it critically for important intellectual content. All provided final approval of the article and agree to be accountable for all aspects of the work.
